# Recurrent Dedifferentiated Clival Chondrosarcoma Presenting 32 Years after Initial Treatment: A Case Report

**DOI:** 10.1055/a-2964-8969

**Published:** 2026-10-06

**Authors:** Pawan Kishore Ravindran, Max E. Keizer, Zeynep B. Erdem, Henricus P.M. Kunst, Koos E. Hovinga, Jasper van Aalst, Yasin Temel

**Affiliations:** 1 Department of Neurosurgery 199236 Maastricht University Medical Center+MaastrichtLimburgThe Netherlands; 2 199236 Dutch Academic Alliance Skull Base Pathology, Maastricht University Medical Center+MaastrichtLimburgThe Netherlands; 3 82246 Mental Health and Neuroscience Research Institute, Faculty of Health, Medicine and Life Sciences, Maastricht UniversityMaastrichtLimburgThe Netherlands; 4 Department of Pathology 4501 Leiden University Medical CenterLeidenThe Netherlands; 5 Department of Ear, Nose and Throat 6034 Radboud Institute of Health Sciences, Radboud University Medical CenterNijmegenGelderlandThe Netherlands; 6 Department of Otolaryngology 199236 Maastricht University Medical Center+MaastrichtLIThe Netherlands; 7 553310 Faculty of Medicine, Istanbul Atlas UniversitesiIstanbulİstanbulTürkiye

**Keywords:** recurrence, IDH1 mutation, chordoma, clival chondrosarcoma, skull base chondrosarcoma, dedifferentiated chondrosarcoma

## Abstract

**Background** Skull base chondrosarcomas are rare and locally aggressive tumors with heterogeneous biology accounting for 0.1–0.2% of all intracranial tumors. Dedifferentiated chondrosarcoma is an exceptionally uncommon and aggressive variant, seldom seen at the skull base. We report a recurrent clival chondrosarcoma with a small, dedifferentiated component, and an unusually long latency after initial treatment.

**Case Report** A 66-year-old woman was referred with a recurrent progressive clival lesion, initially suspected to be a chordoma. She had undergone subtotal resection followed by single-fraction radiosurgery (25 Gy) 32 years earlier. Surveillance imaging demonstrated interval growth compared with imaging 5 years earlier, prompting repeat subtotal resection. Histopathology revealed a conventional chondrosarcoma with a dedifferentiated component comprising <5% of tumor area and an isocitrate dehydrogenase 1 (IDH1) R132 mutation. The patient declined adjuvant radiotherapy. Three years later, she developed worsening diplopia, new right oculomotor palsy, and headache with radiologic progression, prompting repeat subtotal resection. She currently awaits postoperative proton beam radiotherapy.

**Conclusion** This case highlights a key diagnostic challenge of distinguishing chondrosarcoma from chordoma, and underscores the importance of meticulous histologic sampling to detect small dedifferentiated foci that may otherwise result in undergrading and undertreatment. The heterogeneity in clinical behavior observed across histopathological subtypes further underscores the necessity for grade/subtype-driven treatment regimens. Therefore, accurate subtype classification and molecular annotation may better inform treatment strategy and follow-up. Lastly, the rarity of dedifferentiated skull base chondrosarcoma reinforces the need for multicenter registries to further understand the behavior and vulnerabilities of this rare disease.

## Introduction

Chondrosarcomas encompass a heterogeneous group of malignant bone tumors arising from cartilage-producing cells. 1 Chondrosarcoma is the second most common sarcoma, preceded by osteosarcoma, accounting for 20–27% of primary bone malignancies. The majority arise de novo, termed primary chondrosarcoma, while some may develop from preexisting benign lesions, such as enchondromas or osteochondromas, known as secondary chondrosarcomas. 2 Skull base chondrosarcomas form a rare subset of chondrosarcomas, accounting for approximately 2% of cases and 0.1–0.2% of intracranial tumors. 3 4 Skull base chondrosarcomas are thought to arise from cartilaginous remnants at skull base synchondroses and most commonly involve the petroclival region, including the petrous apex and clivus. Skull base chondrosarcomas present at a median age of 43 years (ranging from 18 to 85), with a slight female predominance.

The conventional subtype prevails, and rarer subtypes including clear cell, mesenchymal, and dedifferentiated are also seen. 1 The conventional chondrosarcoma is histologically graded based on cellularity, cytologic atypia/pleomorphism, mitotic activity, and matrix features such as myxoid change. Tumors are classified as grade 1 (low grade, well-differentiated), grade 2 (intermediate grade), and grade 3 (high grade). 5 Molecularly, mutations are noted (e.g., isocitrate dehydrogenase 1 and 2 [IDH1/2] mutations, tumor protein p53 [TP53], phosphatase and tensin homolog [PTEN]); however, the prognostic and therapeutic implications are still under investigation. 6

Conventional skull base chondrosarcomas are considered locally aggressive lesions, with low metastatic propensity. However, the dedifferentiated subtype is characterized by increased aggression and high recurrence rates. 7 Gross total resection is the cornerstone for treating such lesions; however, due to the anatomical localization and proximity to critical neurovascular structures, this is rarely feasible and postoperative radiotherapy is often administered following subtotal resection and/or higher grade disease to provide improved tumor control. 4 7 8 The diverse subtypes and histologic grades of chondrosarcoma exhibit different biological behaviors, prompting consideration of whether treatment approaches should be tailored to tumor biology rather than relying on uniform guidelines for this biologically heterogeneous disease. Here, we present a rare case of recurrent clival chondrosarcoma with a dedifferentiated component and an IDH1 R132 mutation with an exceptionally long latency period after initial subtotal resection and stereotactic radiosurgery, highlighting diagnostic pitfalls, importance of histological subtyping, and implications for therapy.

## Case Report

Our patient is a 66-year-old woman referred to our department with a recurrent clival lesion, initially suspected to be a chordoma. Her past medical history is further notable for melanoma and Hashimoto’s thyroiditis, both clinically stable. Thirty-two years earlier, her clival lesion was treated with subtotal resection followed by single-fraction 25 Gy stereotactic radiosurgery at an outside institution. The original histopathology report was unavailable at presentation to our institution; however, archival tissue was retrieved for retrospective review. The surgery, a microscopic transnasal transsphenoidal approach, was complicated by an internal carotid artery injury, necessitating a subsequent open pterional procedure to achieve hemostasis and further tumor debulking. This was later also complicated by a surgical site infection, from which she recovered fully. She was surveilled with serial magnetic resonance imaging (MRI) scans, and this recurrence was noted 32 years after initial treatment. Clinically, she remained stable and reported no new or progressive symptoms; however, she continued to experience persistent diplopia due to the preexisting left abducens nerve palsy.

MRI of the brain demonstrated ( Fig. 1 ) a well-circumscribed T2-hyperintense clival lesion with heterogeneous contrast enhancement on gadolinium-enhanced T1, extending into the left sphenoid sinus, measuring 16 × 16 × 15 mm (anteroposterior × transverse × craniocaudal), progressive compared to the MRI images 5 years prior (11 × 12 × 14 mm). The case was reviewed in our multidisciplinary skull base conference, involving a neurosurgeon, otolaryngologist, neuroradiologist, and radiation oncologist, and a consensus was reached to proceed with surgical resection. Subtotal resection was performed via an extended endoscopic endonasal approach. Tissue from the clival lesion was sent for histopathological evaluation, including review by our national bone tumor committee. Histopathological evaluation showed two morphological components: conventional chondrosarcoma and a dedifferentiated component ( Fig. 2A ). The conventional component was characterized by chondrocytes embedded in a hyaline to myxoid chondroid matrix, with focal calcification. The tumor showed variable cellularity, cytologic atypia, and increased binucleation of chondrocytes ( Fig. 2B ). In areas showing an abrupt transition from the conventional chondrosarcoma component ( Fig. 2C ), a second component was identified, composed of highly pleomorphic tumor cells with abundant pale eosinophilic cytoplasm set in a collagenized to myxoid matrix, consistent with dedifferentiation ( Fig. 2D ). Mitotic activity was readily identifiable in both components. Immunohistochemically, the neoplastic cells were negative for keratin and brachyury. Molecular analysis demonstrated an IDH1 R132 mutation, supporting the diagnosis of chondrosarcoma. The final diagnosis was reported as dedifferentiated chondrosarcoma, with the dedifferentiated component noted to be limited, comprising less than 5% of the tumor area. Retrospectively, the histopathology of the original tumor in 1991 was reviewed, and no dedifferentiated component was identified ( Fig. 3A,B ). Despite being advised, the patient refused postoperative radiotherapy due to prior treatment-related experiences.

**Fig. 1:**
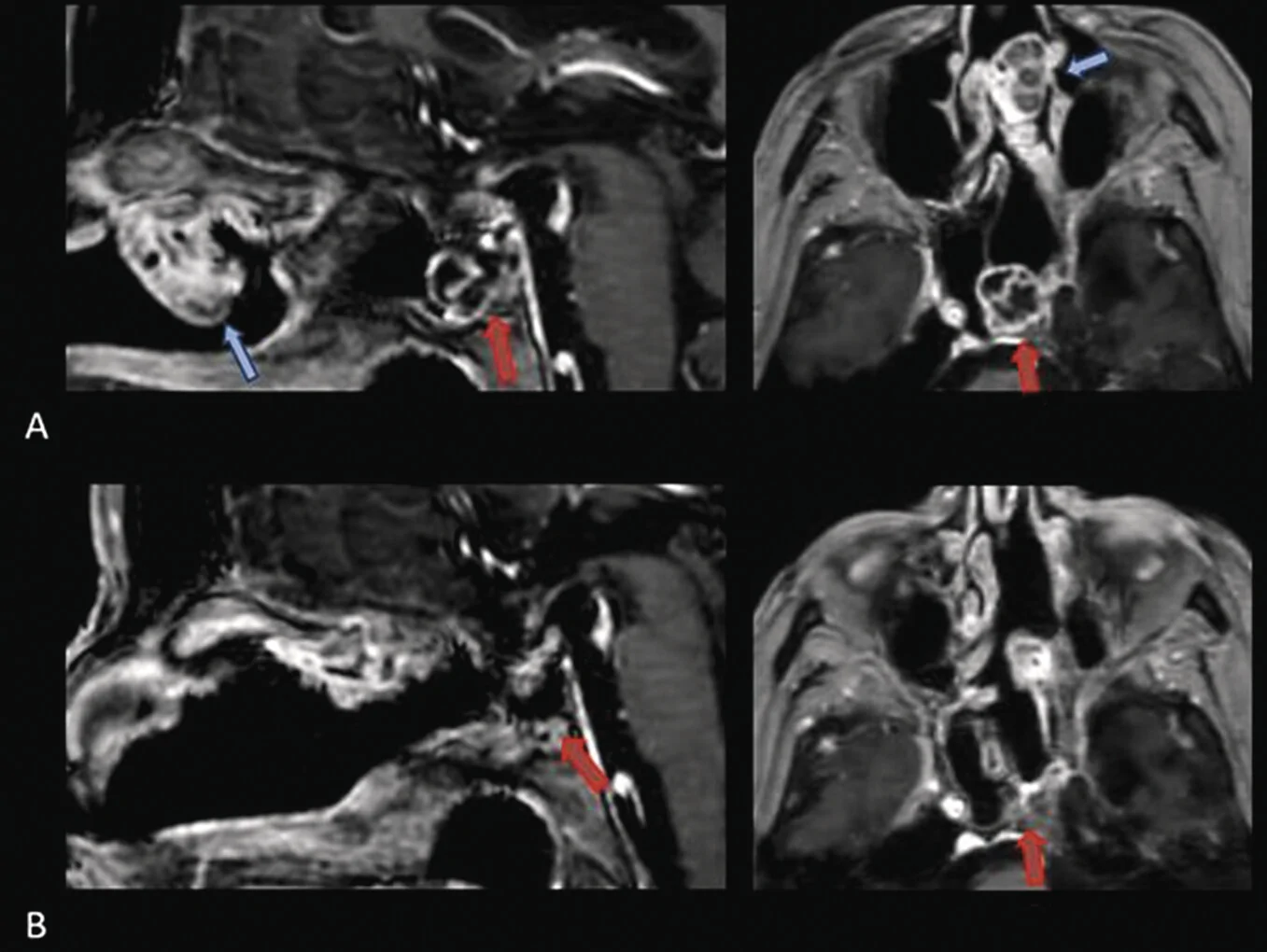
Preoperative and postoperative gadolinium-enhanced T1-weighted MRI of the skull base (sagittal and axial planes). ( **A** ) Preoperative images demonstrate a clival heterogeneous enhancing mass (red arrow). A separate lesion in the left middle turbinate is also shown (blue arrow) and was confirmed to be benign (non-neoplastic). ( **B** ) Postoperative images following extended endoscopic endonasal resection show the surgical resection cavity at the clivus (red arrow). MRI, magnetic resonance imaging.

**Fig. 2:**
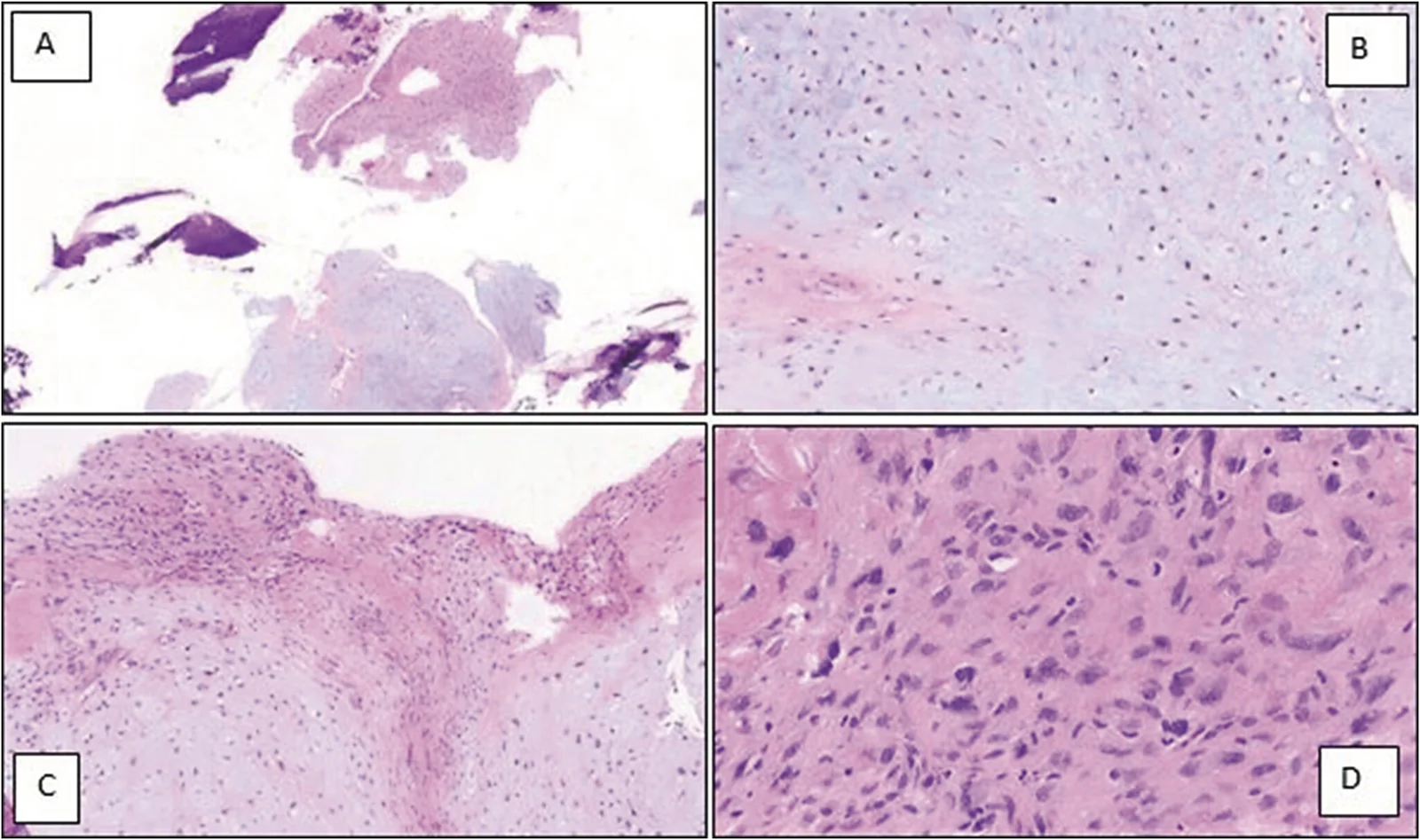
( **A** ) Low-power histology image showing two morphologically distinct components: conventional chondrosarcoma (lower fragment) and a dedifferentiated component (upper fragment; H&E, ×5). ( **B** ) Conventional chondrosarcoma composed of chondrocytes with variable cellularity and atypia, embedded in a myxoid to hyaline chondroid matrix (H&E, ×20). ( **C** ) Abrupt transition from the conventional chondrosarcoma component to areas of dedifferentiation (H&E, ×10). ( **D** ) Dedifferentiated component composed of spindle to pleomorphic tumor cells in a collagenized to myxoid matrix (H&E, ×40). H&E, hematoxylin and eosin.

**Fig. 3:**
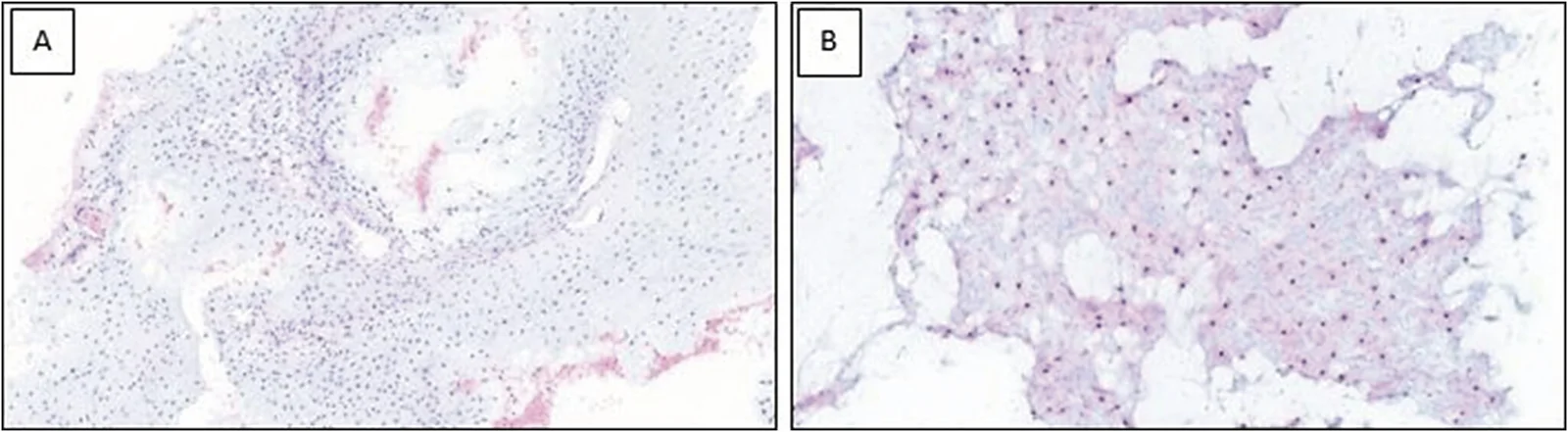
( **A, B** ) The primary tumor showed a low- to moderately cellular neoplasm with minimal cytologic atypia and occasional binucleated cells. No dedifferentiated component was identified in the evaluated material. (H&E; ×10, ×20). H&E, hematoxylin and eosin.

Our patient remained stable for the next 3 years, after which she had episodes of worsening diplopia, a new paresis of her right oculomotor nerve, and headache. A repeat MRI of the brain was made, which revealed progression; the tumor now measured 22 × 18 × 24 mm ( Fig. 4A ). She underwent repeat subtotal resection via an extended endoscopic endonasal approach. Postoperatively, her headaches resolved, the cranial nerve palsies still persisted, and her diplopia worsened. The postoperative MRI scan showed reduction in volume of the tumor component extending into the left sphenoidal sinus ( Fig. 4B , red arrow), with interval increase in size of the tumor remnant paramedian on the right in the sella turcica/cavernous sinus, now measuring 18 × 20 mm, previously 13 × 14 mm ( Fig. 4B , blue arrow). At the time of writing, she is scheduled to receive postoperative proton beam radiotherapy.

**Fig. 4:**
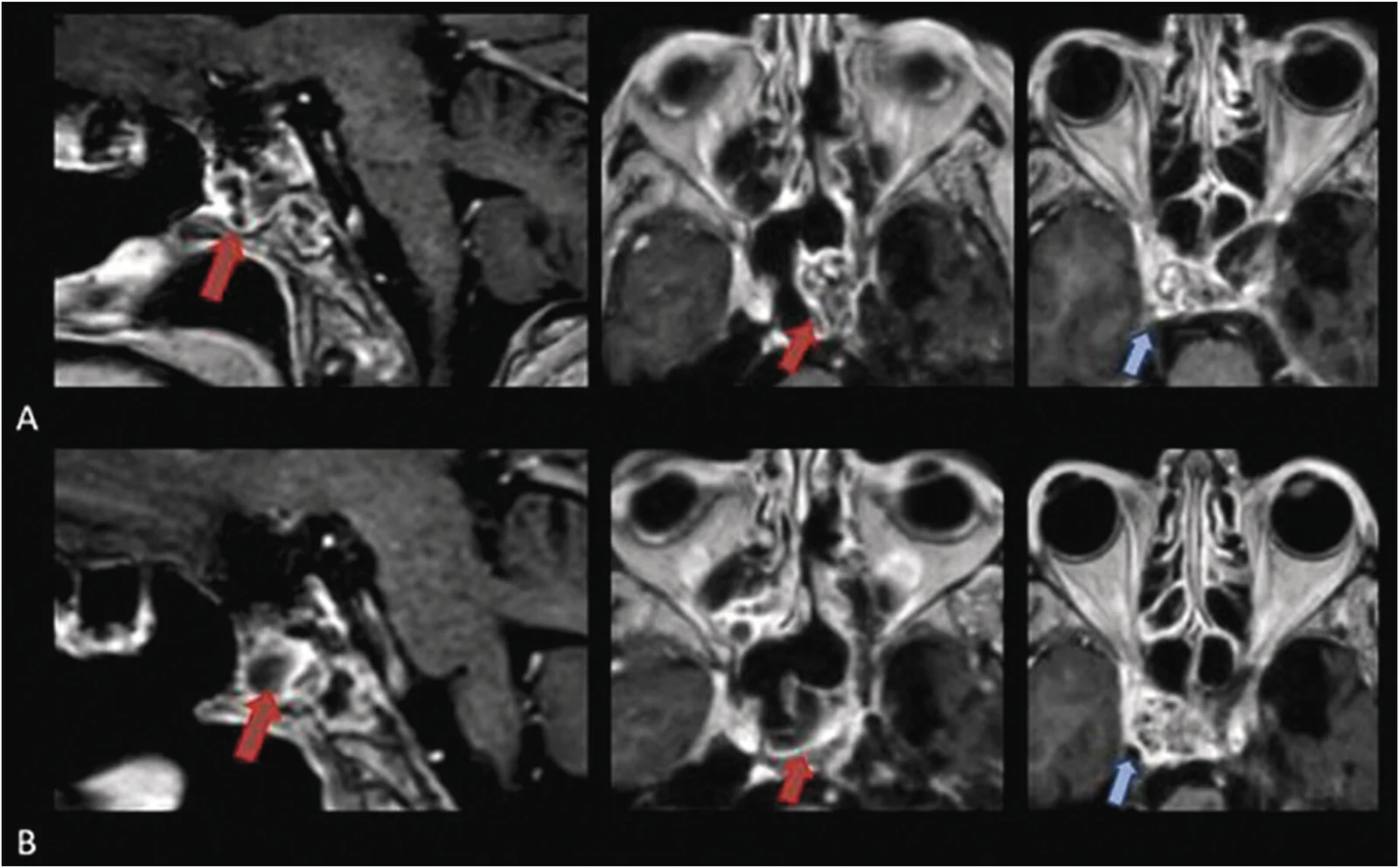
Gadolinium-enhanced T1-weighted MRI of the skull base at the time of recurrence ( **A** ) and postoperatively ( **B** ). ( **A** ) Preoperative sagittal and axial postcontrast T1-weighted images show a heterogeneously enhancing lesion centered at the left petroclival region with extension towards the sphenoid sinus and cavernous sinus (red arrows). A smaller component is noted in the sella/cavernous sinus (blue arrow). ( **B** ) Postoperative images following tumor debulking demonstrate reduction in the intrasinusal tumor component (red arrows). However, there is interval progression of a peripherally enhancing tumor remnant in the sella/cavernous sinus (blue arrow). MRI, magnetic resonance imaging.

## Discussion

This case report describes the clinical course of a 66-year-old woman with dedifferentiated chondrosarcoma, a rare subtype of an already uncommon skull base malignancy. The first challenge was the initial diagnosis of clival chordoma. Nonetheless, this is a well-recognized pitfall, as both chordomas and chondrosarcomas can share the same anatomic location, clinical presentation, and radiological characteristics—resulting in their diagnostic overlap and historical misclassification, both in clinic and literature. The differentiating factor between these rare tumors, both of which share a combined incidence of 0.03 per 100,000, lies in their origin and histological and immunohistochemical features. 9

Skull base chordomas arise from remnants of the notochord, whereas chondrosarcomas originate from primitive mesenchymal cells or from embryonic rests of the cartilaginous matrix of the skull base. Chordomas come in three histological subtypes, namely, conventional, poorly differentiated, and dedifferentiated. 10 Histologically, conventional chordomas have a lobulated architecture separated by fibrous bands. Within the lobules, tumor cells are arranged in cords and short strands, as well as nests and scattered single cells. These epithelioid tumor cells have a vacuolated appearance, termed physaliphorous cells; characteristic of chordoma. The nuclei are typically vesicular with variable pleomorphism. 11 Chondroid chordoma, a subtype of the conventional chordoma, also contains a cartilaginous component. Chordomas stain positive for cytokeratins/epithelial membrane antigen, S100, and brachyury. Chondrosarcomas are brachyury negative and typically non-reactive for keratin antigens and epithelial membrane antigen. 12 The role of brachyury as a diagnostic hallmark and oncogenic driver was established in the mid-2000s; as it entered routine diagnostic practice relatively recently, historical cases, such as ours, may have been vulnerable to misclassification due to the absence of modern markers. 13 14

The high-grade dedifferentiated subtype accounts for 10% of all chondrosarcomas and is typified by two distinctive histopathological components: a well-differentiated cartilaginous component composed of chondrocyte-like neoplastic cells intersected with a high-grade dedifferentiated component, characterized often with spindle-shaped neoplastic cells. 15 16 17 This dedifferentiated component may be small; in our case, it accounted for <5% of the tumor, thus pathologists should sample cartilaginous tumors thoroughly and scan for grossly abnormal zones. This rare subtype is typically seen in older adults with a median age of 60 years. Dedifferentiated chondrosarcoma can either occur spontaneously or develop from a preexisting low-grade chondrosarcoma. 16 In our patient, progression of the original tumor is a plausible explanation given the same anatomical location, prior subtotal resection, radiological surveillance that occurred during the 32 years and the absence of a dedifferentiated component on retrospective review of the 1991 histopathology. However, the possibility of an unsampled dedifferentiated focus or a second primary tumor cannot be ruled out.

Given the history of stereotactic radiosurgery 32 years earlier, a potential contribution of prior irradiation to the subsequent dedifferentiation cannot be entirely excluded. Chondrosarcoma is particularly resistant to conventional radiotherapy due to several biological factors, including its slow proliferation, the dense extracellular matrix, stromal cells, cancer stem cells, and hypoxic microenvironment. 18 Although radiation-induced sarcomas, including de novo chondrosarcoma and dedifferentiated chondrosarcoma, have been reported, 19 20 evidence that irradiation can induce dedifferentiation of a preexisting conventional chondrosarcoma is lacking. The prolonged latency between radiotherapy and tumor presentation parallels that reported in case reports of radiation-induced chondrosarcomas, suggesting a potential role of prior irradiation. Notably, in the series published by Breshears et al, two patients had an increase in histologic grade from conventional grade II to grade III, both of whom had received radiotherapy prior to transformation. 21 While these observations do not establish causality, they raise the possibility that prior irradiation may influence tumor evolution or select for more aggressive cell populations. Further investigation into the biological alterations occurring in chondrosarcoma following radiotherapy is warranted.

The risk of this dedifferentiation is reported to be 7–20%; however, what triggers this development remains under investigation. 16 Sanerkin and Woods proposed a collision model, in which two separate neoplastic clones develop in parallel from a multipotent mesenchymal precursor and give rise to histologically distinct tumor populations. 22 In contrast, most molecular evidence favors a monoclonal origin, whereby both components descend from a single primitive mesenchymal cell, and the high-grade dedifferentiated component emerges through genomic instability and acquisition of additional alterations. 15 Several biological alterations have been suggested, including mutations in TP53, cyclin-dependent kinase inhibitor 2A/2B (CDKN2A/B), collagen type II alpha 1 chain (COL2A1), PTEN, retinoblastoma protein-E2F transcription factor (RB-E2F) pathway, neuroblastoma RAS viral oncogene homolog (NRAS), and telomerase reverse transcriptase (TERT). 15 Our patient harbored an IDH1 R132 mutation, the most common variant of IDH1/2 mutation found in chondrosarcomas. 23 IDH mutations confer a neomorphic enzymatic activity that converts α-ketoglutarate (α-KG) to the oncometabolite D-2-hydroxyglutarate (D-2HG). Accumulation of D-2HG competitively inhibits α-KG-dependent dioxygenases, leading to broad alterations in epigenetic regulation, DNA repair, and cell growth signaling, as well as rewiring of cellular metabolism. 23 This mutation is considered to play a significant role in the early stages of tumor development; however, the prognostic implication of IDH mutations in chondrosarcoma remains unclear, as studies show contradictory results. 23 Furthermore IDH1-mutant inhibition shows mixed results, some in vitro studies report little impact on tumorigenic properties, whereas others report reduced proliferation with high doses or different inhibitors. Collectively, the underlying tumor biology of dedifferentiated chondrosarcoma remains incompletely understood and represents a potential avenue for the development of targeted and systemic therapies.

Dedifferentiated chondrosarcoma of the skull base represents an exceptionally rare entity, with the existing literature largely limited to isolated case reports. The management of skull base dedifferentiated chondrosarcoma is identical to that of a high-grade chondrosarcoma, necessitating a multimodal approach, namely maximal safe resection followed by adjuvant high-dose radiotherapy. 4 The role of radiotherapy as a primary therapy remains controversial as chondrosarcomas are relatively resistant to radiotherapy. Currently, proton therapy is used, primarily due to the Bragg peak phenomenon and the ability to relatively spare the organs at risk. However, with the development of various radiation delivery techniques, photon therapy is becoming an acceptable alternative. 4 Generally, chondrosarcomas are resistant to chemotherapy; however, some institutions do utilize chemotherapy for localized and metastatic dedifferentiated chondrosarcoma, often following protocols for osteosarcoma; however, the benefit is debated. 7 16 Despite aggressive multimodal therapy, skull base chondrosarcomas have progression-free survival rates across series ranging from 87.0% to 96.2% at 3 years, 57.1% to 100% at 5 years, and 67.0% to 100% at 10 years. Due to the rarity of skull base dedifferentiated chondrosarcoma, histology-specific outcome data are limited. In one of the largest histology-stratified skull base chondrosarcoma series available, mesenchymal ( *n* = 6) and dedifferentiated ( *n* = 1) chondrosarcomas were grouped together and showed markedly worse median progression-free survival (24 months) compared with conventional tumors (166 months), with outcomes strongly influenced by extent of resection. 4 7

In summary, this case underscores the diagnostic and therapeutic implications of histologic subtype in skull base chondrosarcomas. Notably, the exceptionally long latency between initial treatment and subsequent progression suggests that skull base chondrosarcomas may recur or undergo malignant transformation even decades after initial management, supporting prolonged surveillance. Also, the dedifferentiated component constituted <5% of the tumor, emphasizing the importance of extensive histologic sampling in recurrent or progressive cartilaginous lesions to avoid undergrading. Given the markedly more aggressive course of dedifferentiated chondrosarcoma compared with conventional disease, accurate classification and adequate sampling are essential to guide treatment intensity, radiotherapy strategy, and follow-up. Greater representation of rare subtypes in multicenter registries and molecularly annotated series may enable improved prognostication and the development of tailored therapeutic approaches.
